# Antibody Response to COVID-19 Booster Vaccination in Healthcare Workers

**DOI:** 10.3389/fimmu.2022.872667

**Published:** 2022-05-26

**Authors:** Arianna Pani, Alessandra Romandini, Alice Schianchi, Michele Senatore, Oscar M. Gagliardi, Gianluca Gazzaniga, Stefano Agliardi, Tommaso Conti, Paolo A. Schenardi, Matteo Maggi, Stefano D’Onghia, Valentina Panetta, Silvia Renica, Silvia Nerini Molteni, Chiara Vismara, Daniela Campisi, Michaela Bertuzzi, Simona Giroldi, Laura Zoppini, Mauro Moreno, Marco Merli, Marco Bosio, Massimo Puoti, Francesco Scaglione

**Affiliations:** ^1^Oncology and Hemato-Oncology Department, Università degli Studi di Milano, Milan, Italy; ^2^Chemical-Clinical Analysis Unit, Laboratory Medicine Department, Niguarda Hospital, Milan, Italy; ^3^Biostatistics Office, L’altrastatistica srl – Consultancy & Training, Rome, Italy; ^4^Microbiology Unit, Laboratory Medicine Department, Niguarda Hospital, Milan, Italy; ^5^Quality & Risk Control in Data Management, Azienda Socio Sanitaria Territoriale (ASST) Grande Ospedale Metropolitano Niguarda, Milan, Italy; ^6^Socio-sanitary Direction, Azienda Socio Sanitaria Territoriale (ASST) Grande Ospedale Metropolitano Niguarda, Milan, Italy; ^7^Healthcare Management Department, Azienda Socio Sanitaria Territoriale (ASST) Grande Ospedale Metropolitano Niguarda, Milan, Italy; ^8^Infectious Diseases Unit, Azienda Socio Sanitaria Territoriale (ASST) Grande Ospedale Metropolitano Niguarda, Milan, Italy; ^9^School of Medicine and Surgery, Università degli Studi di Milano Bicocca, Milan, Italy

**Keywords:** COVID-19, SARS-CoV-2, vaccine booster, healthcare workers (HCWs), antibody response

## Abstract

**Objective:**

To evaluate the mean increase of anti-S IgG antibody titer between the basal, pre-booster level to the titer assessed 14 days after the booster dose of BNT162b2.

**Patients and Methods:**

The RENAISSANCE study is an observational, longitudinal, prospective, population-based study, conducted on healthcare workers of Niguarda Hospital in Milan, Italy who received a BNT162b2 booster dose at least 180 days after their second dose or after positivity for SARS-CoV-2 and accepted to take part in the study. The RENAISSANCE study was conducted from January 1, 2021 through December 28, 2021.

**Findings:**

1,738 subjects were enrolled among healthcare workers registered for the booster administration at our hospital. Overall, 0.4% of subjects were seronegative at the pre-booster evaluation, and 1 subject had a titer equal to 50 AU/ml: none of the evaluated subjects was seronegative after the booster dose. Thus, the efficacy of the booster in our population was universal. Mean increase of pre- to post-booster titer was more significant in subjects who never had SARS-CoV-2 (44 times CI 95% 42-46) compared to those who had it, before (33 times, CI 95% 13-70) or after the first vaccination cycle (12 times, CI 95% 11-14). Differently from sex, age and pre-booster titers affected the post-booster antibody response. Nevertheless, the post-booster titer was very similar in all subgroups, and independent of a prior exposure to SARS-CoV-2, pre-booster titer, sex or age.

**Conclusion:**

Our study shows a potent universal antibody response of the booster dose of BNT162b2, regardless of pre-booster vaccine seronegativity.

## Introduction

The dynamic evolution of the COVID-19 pandemic situation has so far driven an unprecedented program of rolling review and instant evaluation of drugs by national and international regulators.

Following this line, on August 12, 2021 the FDA amended the emergency use authorization for the mRNA vaccines (BNT162b2 and mRNA-1273) to allow the use of a booster dose for immunocompromised people. On the other hand, EMA concluded that an extra dose of the mRNA vaccines may be given to people with severely weakened immune systems or whose frequent exposure puts them at high risk for severe COVID-19, at least 28 days after their second dose, though individual European countries approved the booster vaccine for different indications.

This was dictated by the emergence of new variants of concern, such as the delta variant (1) and the omicron variant (1, 2), the resurgence of confirmed infection as well as severe illness in both vaccinated and unvaccinated subjects, though in very different rates (3–5), and the risk of new nation-wide restrictions and lockdowns.

So far, there have been few studies on the serologic response to the booster dose and have mainly been population-specific: onco-hematologic patients (6, 7), patients receiving hemodialysis or peritoneal dialysis (8), immunocompromised patients (9), heart or kidney transplant recipients (10, 11). Moreover, on November 8, 2021, the Omicron variant was first sequenced and identified in South Africa (1, 2) and caused a quick, significant resurgence in SARS-CoV-2 cases internationally. As this resurgence sparked doubts on vaccines’ efficacy, Pfizer issued a claim stating that early laboratory tests show that sera from individuals who received three doses of the BNT162b2 vaccine neutralize the Omicron variant, while two doses exhibit more than a 25-fold reduction in neutralization titers against the Omicron variant compared to wild-type, indicating that two doses may not be sufficient to protect against infection with the Omicron variant (12).

RENAISSANCE (13) is an observational, longitudinal, prospective, population-based study on healthcare workers (HCWs) of Niguarda Hospital in Milan, Italy, evaluating the antibody response to the BNT162b2 vaccine in staff members of one of the main COVID-19 reference hospital of Milan, Italy, at multiple time points after a hospital-wide vaccination campaign.

With the indication to administer a booster vaccine to HCWs at Niguarda Hospital in Milan, Italy, the RENAISSANCE study included an additional time point before and after the booster dose of the vaccine to evaluate the serologic response to it.

## Materials and Methods

### Objectives, Participants, and Oversight

All staff who had completed the BNT162b2 vaccine schedule by March 7, 2021 at Niguarda Hospital and who received a booster dose of BNT162b2 between October 11 and November 12, 2021 were eligible for inclusion. Subjects had been vaccinated with two doses of BNT162b2 of 30 µg 21 days apart as described in the summary of product characteristics (14), regardless of prior history of SARS-CoV-2 infection and received a booster dose at least 5 months after their second dose or, if infected after the vaccination, after the positivity for SARS-CoV-2.

The primary endpoint of the study was the evaluation of the mean increase of anti-RBD IgG antibody titer between the basal, pre-booster level to the titer assessed 14 days after the booster dose of BNT162b2.

Subjects were classified as either having had a positivity for SARS-CoV-2 or never having had contact with the virus. The criteria used to identify subjects who had had a prior positivity for SARS-CoV-2 before receiving the booster were:

- previous RT-PCR positive during the surveillance program;
- previous anti-Nucleocapsid (N) total IgG seropositivity during the 14 days, 3-months and 6-months follow-up of the RENAISSANCE study (13);
- recent anti-N IgG positivity at the baseline during the third dose follow-up.

### Procedures

The anti-COVID-19 booster vaccination program at Niguarda Hospital started on October 11^th^, 2021 and rolled out until November 12^th^, 2021. All hospital staff were invited receive the BNT162b2 booster by email.

Upon administration, the RENAISSANCE study team invited the subjects to participate in the study.

One sample of venous blood was drawn from each participant right before the booster administration, and one 14 (-2, +7) days after it. Both blood samples were stored at room-temperature upon collection, and then centrifuged to separate plasma, which was conserved at +4°C until processing, which occurred within 30 days.

### Laboratory Methods

SARS-CoV-2 anti-RBD IgG antibodies titers were assessed through the SARS-CoV-2 IgG II Quant, an automated, two-step chemiluminescent microparticles immunoassay (CMIA) able to detect the presence of antibodies for the receptor-binding domain (RBD) on the S1 subunit of the Spike glycoprotein, intended for use on the Alinity i. This test is manufactured by Abbott^®^, and is a qualitative and quantitative test with optimal sensitivity and specificity for IgG detection, and a 100% correlation with neutralizing antibodies titers (IC 95%, 95.72-100.00) (15).

Results are provided as arbitrary units per milliliter (AU/mL), and values <50.0 are reported as negative, accordingly to the manufacturer’s indications. The test is able to detect titers up to 40,000 AU/mL (15). Seronegativity was defined as having a titer <50.0 AU/ml.

Total anti-N IgG antibodies titers were assessed through the SARS-CoV-2 IgG by Abbott^®^, an automated, two-step CMIA, able to detect antibodies for the nucleocapsid protein of SARS-CoV-2, intended for use on the Alinity i. The signal/cut-off (S/C) ratio <1.4 is interpreted as negative, while a S/C ratio ≥ 1.4 was interpreted as reactive.

The sensitivity and specificity of the used tests is not indicated in the summary of product characteristics, though the qualitative test performance was evaluated through the Positive Percent Agreement (PPA) and the Negative Percent Agreement (NPA). The SARS-CoV-2 IgG II Quant PPA evaluated against PCR reached 99.35% (CI 95%, 96.44-99.97) for patients with symptoms onset at least 15 days prior, with lower percentages for patients with more recent symptom onset. Its NPA was 99.60% (CI 95%, 99.22-99.80) (15).

The anti-N SARS-CoV-2 IgG test reached a PPA of 100% (CI 95%, 95.89-100.00) and a NPV of 99.63 (CI 95%, 99.05-99.90).

### Statistics

Categorical variables are summarized as number (n) and percentage (%). Age was summarized as mean and standard deviations (sd) and was also presented in classes (19-30yo; 31-40yo; 41-50yo; 51-60yo; >60yo).

To evaluate the pre- and post-booster change in antibodies values (AU/ml) a tobit mixed model regression was used taking in account the maximum detectable value by machine (40,000 AU/ml).

In all models the dependent variable was the log 10 of antibodies and ID of participants was considered as random factor.

In the first model the fixed factors were the interaction between time (pre-/post-booster) and the history of prior SARS-CoV-2 infection. The subsequent models were only developed in subjects without prior SARS-CoV-2 infection. The fixed factor was the interaction between time and sex in one model, the interaction between time and class of age in the other model and the interaction between time and antibodies classes at pre-booster in the third model. Another tobit model was implemented, considering the interaction between time and sex adjusted for age (as continuous variable). Geometric mean at each timepoint and the titer increase (with their CI 95%) were estimated from the models.

Median and first and third quartile (Q1-Q3) for history of prior SARS-CoV-2 at pre- and post-booster timepoints were also reported.

Contrast Stata command (that performs Wald test) was used after each regression for comparison.

Antibodies were classified based on quartile values of the group of subjects naïve for SARS-CoV-2 infection.

A p value < 0.05 was considered statistically significant. Stata 16.1 was used for all statistical analysis.

### Study Approval

The RENAISSANCE study was approved through joint approval procedure by the AIFA (Italian Agency for Pharmaceuticals) and Spallanzani Hospital, Rome on 19^th^ of January 2021. The approval was registered as n. 253 in the Trials Registry.

## Results

### Study Population

1,738 subjects were enrolled, among whom 1,425 never had a contact with the virus while 313 were classified as having had a SARS-CoV-2 positivity according to previous RT-PCR positivity or to anti-N IgG positivity. We further classified the subgroup of SARS-CoV-2 infected subjects according to the timing of infection: 301 subjects had a history of SARS-CoV-2 infection before the vaccination schedule, 12 after the first or the second vaccine dose. All subjects who had prior SARS-CoV-2 infection, except for one who was infected with SARS-CoV-2 after the first dose, had received two doses.

**Figure 1:**
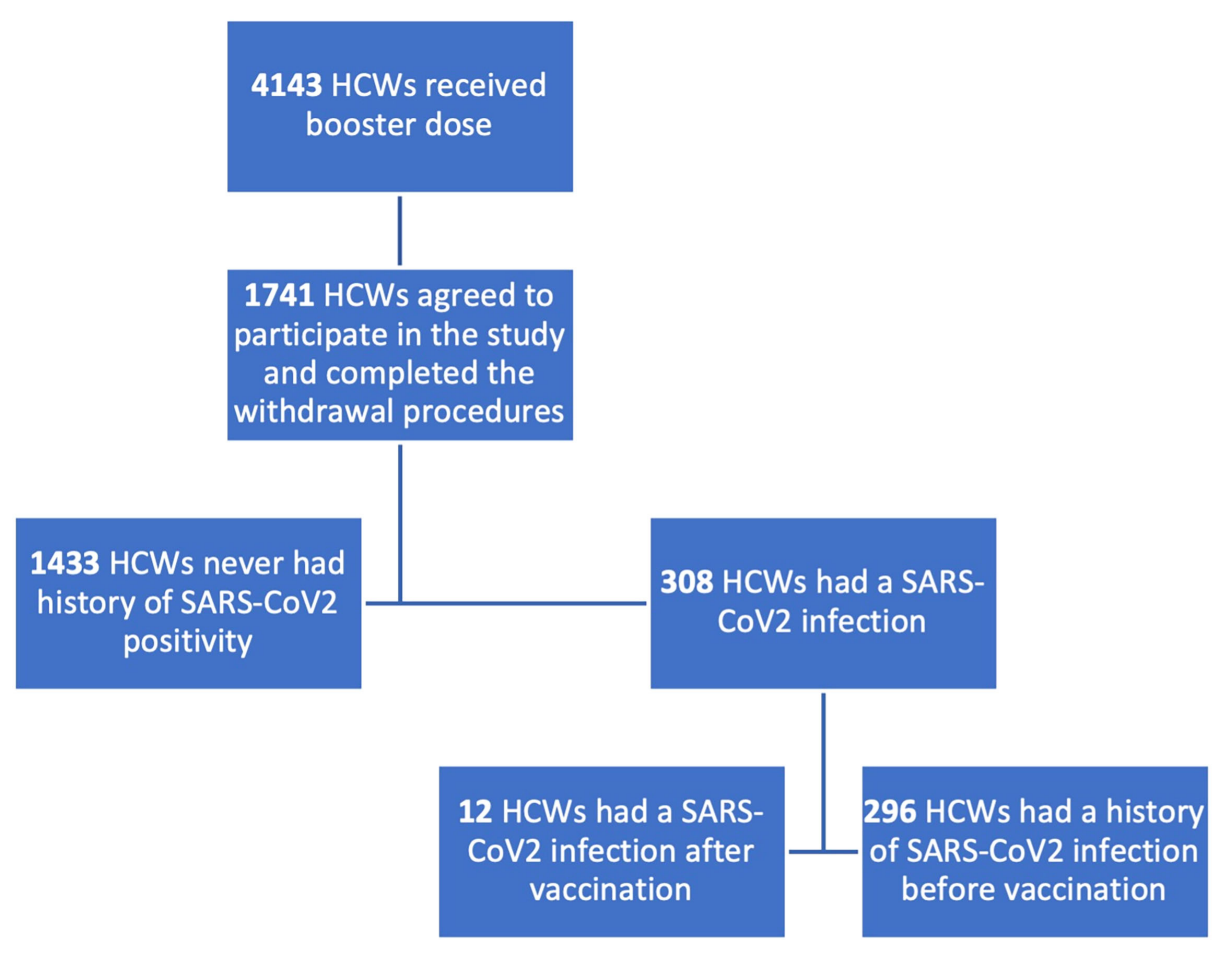
Flowchart describing enrolment process and population classification. HCWs, healthcare workers. 1,738 subjects were enrolled in the booster study, 1425 were naïve for SARS-CoV2 infection, 313 had a history of contact with the virus.

In **Figure 1**, the enrolment process and the population classification according to SARS-CoV-2 positivity are described.

**Table 1** shows the population distribution according to age and sex.

**Table 1:** Population characteristics.

		No SARS-CoV-2 infection		SARS-CoV-2 infection before vaccine		SARS-CoV-2 infection after vaccine		Total	
N		1433		296		12		1741	
Sex (n,%)									
	Female	952	66.4	193	65.2	9	75.0	1154	66.3
	Male	481	33.6	103	34.8	3	25.0	587	33.7
Age (mean sd)		47.37	11.21	45.85	11.1	43.19	12.58	47.04	11.22
Age (n,%)									
	19-30y	118	9.6	31	10.5	2	16.7	151	9.9
	31-40y	231	18.9	63	21.3	3	25.0	297	19.4
	41-50y	276	22.5	80	27.0	3	25.0	359	23.4
	51-60y	463	37.8	96	32.4	3	25.0	562	36.7
	>60y	136	11.1	26	8.8	1	8.3	163	10.6

401 (23%) subjects had post-booster titers above the maximum titrating ability of the test, > 40,000 AU/mL. In particular, 19% (56) of those who had SARS-CoV-2 infection before the first vaccination cycle, 33% (4) of those who had the infection during or after the first vaccination cycle (two doses), and 24% (341) of those who never had the infection.

Overall, 7 out of 1,738 (0.4%) subjects were seronegative at the pre-booster evaluation after a period of 7-9 months from the first vaccination cycle, and 1 subject had a titer equal to 50 AU/ml. None of the evaluated subjects was seronegative after the booster dose. The minimum titer in the whole population was value 1,972 AU/ml, while in the seronegative HCWs group at pre-booster evaluation, the post-booster titer ranged from 2,232 to 40,000 AU/ml. Titers of subjects who were naïve to SARS-CoV-2 infection ranged between 1,972 and 40,000 AU/ml, titers of subjects who had had SARS-CoV-2 infection before the first cycle of vaccination ranged from 2,568 to 40,000 AU/ml, while titers of subjects who had SARS-CoV-2 infection during or after the first cycle of vaccination ranged between 6,142 and 40,000 AU/ml.

### Pre and Post Booster Anti-RBD IgG Antibody Titer

**Figure 2:**
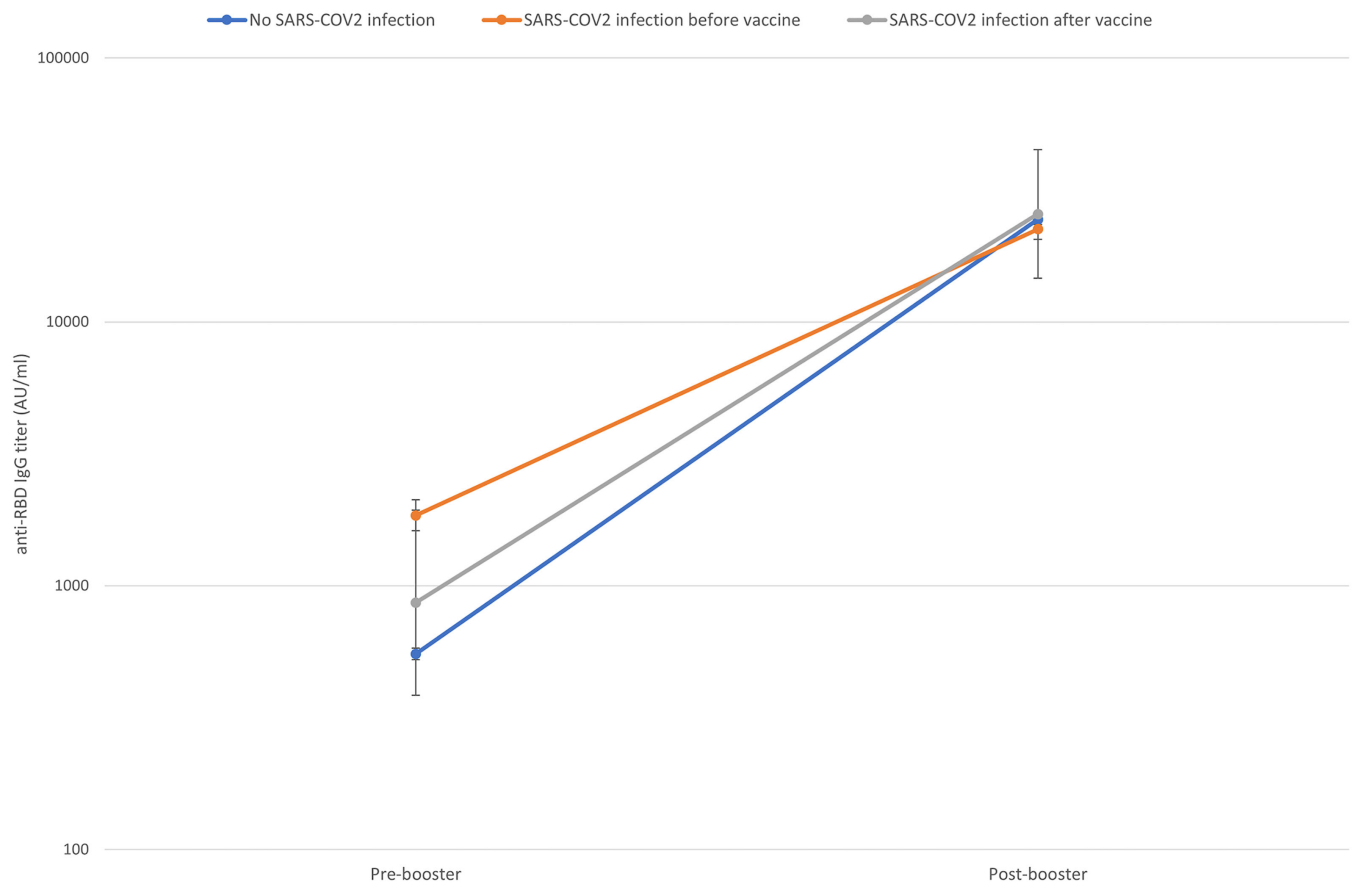
Pre to 14 days post-booster titre increase according to previous SARS-CoV-2 contact (naïve subjects in blue, subjects who had a SARS-CoV-2 infection before the first vaccination cycle in orange, subjects who had a SARS-CoV-2 infection after the first vaccination cycle in grey). Geometric means and CI95% were estimated from tobit mixed model regression on log 10 antibodies.

**Figure 2** shows the titer increase from the pre-booster level to the 14 days post-booster level according to the SARS-CoV-2 infection status.

As shown in **Table 2**, the pre-booster titer evaluated at 285 (IQR 280-290) days from the first dose was higher in HCWs with a history of SARS-CoV-2 infection before the first dose (anti-RBD IgG GM 1,836 AU/ml, 95% CI 1,603-2,103) compared to those with no history of SARS-CoV-2 infection (anti- RBD IgG GM 555 AU/ml, 95% CI 528-582) and those with an infection occurred during or after first vaccination course (anti-RBD IgG GM 864 AU/ml, 95% CI 385-1,938). The mean increment of the anti-RBD IgG was 44 times (CI 95% 42-46) in subjects who never had contact with SARS-CoV-2 virus, 30 times (CI 95% 13-70) in those infected during or after the first vaccination course and 12 times CI 95% 11-14) in subjects infected before vaccination.

**Table 2:** Anti-RBD IgG Geometric mean titre before and after booster vaccination according to previous SARS-CoV2 infection status.

	No SARS-CoV-2 infection			SARS-CoV-2 infection before vaccine			SARS-CoV-2 infection after vaccine			Total		
	GM	CI95%		GM	CI95%		GM	CI95%		GM*	CI95%*	
Pre-booster	555	528	582	1836	1603	2103	864	385	1938	682	651	715
Post-booster	24460	23405	25560	22244	20361	24301	25646	14643	44915	24076	23136	25056
	Mean	IQR		Mean	IQR		Mean	IQR		Mean	IQR	
Pre-booster	522	300	942	1868	820	45505	1035	334	2809	615	322	1238
Post-booster	23525	14381	39200	21670	13475	32762	27350	1738	40000	23102	14226	38100

*Estimated from mixed model.

GM, geometric mean; CI, confidence interval; IQR, interquartile range.

Due to low sample size in the subjects infected during or after vaccinations, statistical comparisons were made only between the group of subjects who never had the infection and the group of subject infected before. The pre-booster titer was significantly higher in subjects who contracted the SARS-CoV-2 infection, with a log10 difference of 0.53 AU/ml, CI 95% 0.46-0.59 p < 0.001, while there is an inversion in the post-booster titer. Post-booster titer was 8% lower in the subgroup of subjects with a previous SARS-CoV-2 infection: the log10 difference is -0.037 (CI 95% -0.08;0.01) p = 0.085.

### Antibody Response According to Sex and Age

We evaluated the impact of age and sex for the group of subjects naïve for SARS-CoV-2 infection.

Pre-booster titers in males and females are at the limits of significance: the log10 difference is -0.042 AU/ml (CI 95% -0.09; -0.00) p = 0.073, where men experience a titer increase of 10%. In contrast to this difference, post-booster titers are similar (p = 0.584). The average increase is 42 times (CI 95% 40-45) for females and 48 times (CI 95% 44-52) for males (p = 0.019). **Figure 1S** illustrates the increase trend from pre- to post-booster titer according to sex.

Adjusting the model for age, there was no difference between male and female subjects either in the pre-booster titers (p = 0.103) or in the post-booster (p = 0.466) and age was negatively correlated to titer. Mean increase is 42 times (CI 95% 40-45) for female subjects and 47 times (CI 95% 44-52) for males (p = 0.021).

We divided our population in 5 classes of age (19-30, 31-40, 41-50, 51-60, >60 years). Even according to class of age, our data shows some differences in pre-booster titers while post-booster titers are similar, as shown in **Table 3S**.

The mean increase is 29 times (CI 95% 26-34) for the class 18-30 years, 38 times (CI 95% 35-43) for the class 31-40 years, 47 times (CI 95% 42-52) for the class 41-50 years, 49 times (CI 95% 46-53) for the class 51-60 years and 51 times (CI 95% 45-59) for the class >60 years.

### Antibody Response According to Pre-Booster Titer

We evaluated the impact of pre-booster antibody levels for the group of subjects naïve for SARS-CoV-2 infection.

**Figure 3:**
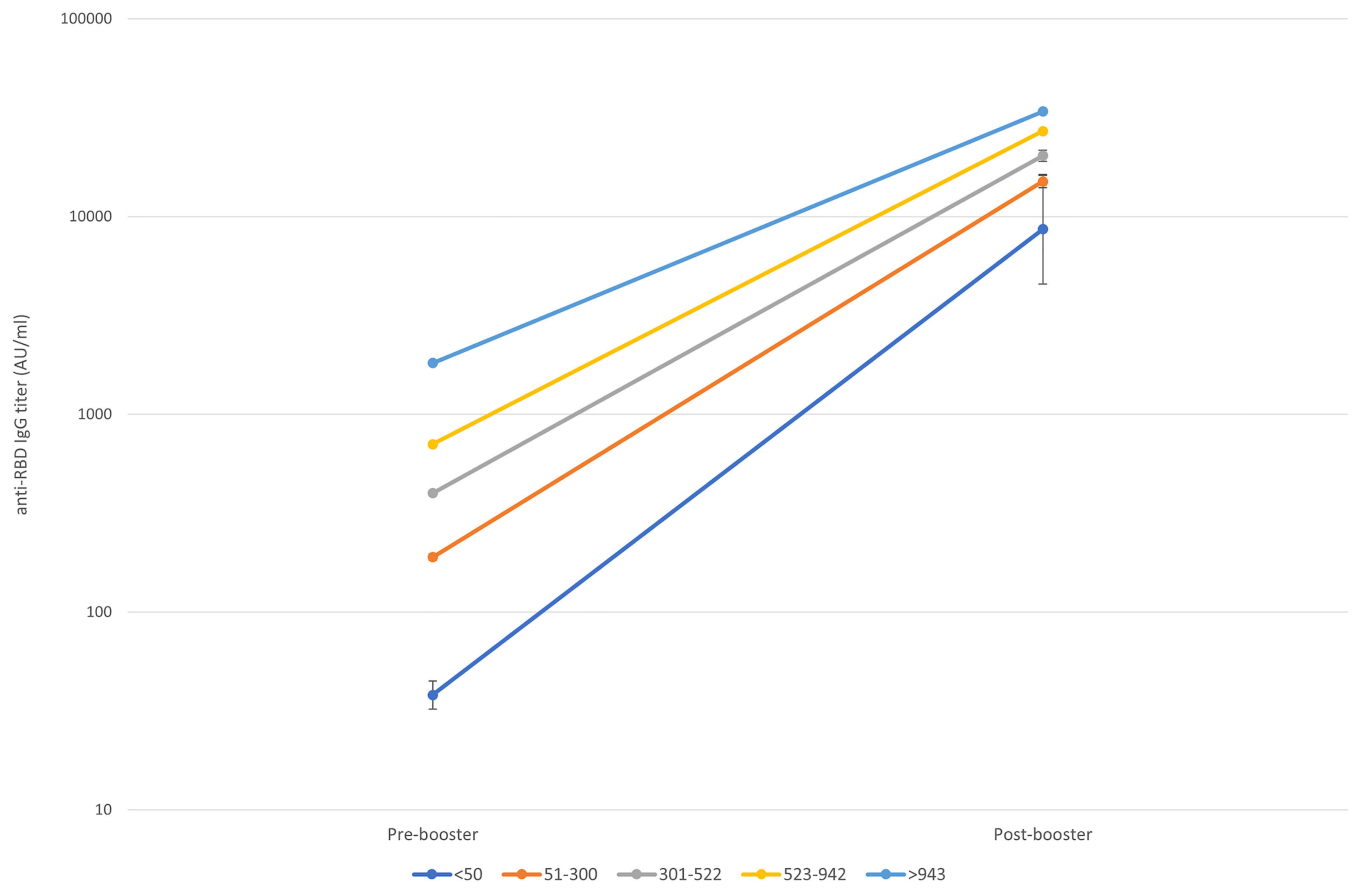
Pre to 14 days post-booster titre increase according to the starting titre. Geometric means and CI95% were estimated from tobit mixed model regression on log 10 antibodies.

**Figure 3** shows the trend of the response according to 5 classes of pre-booster antibody titers basing on quartiles values (<50, 51-300, 301-522, 523-942, >943 AU/ml).

Although the post-booster titers vary depending on the pre-booster titers, we noted a different increasing trend in each pre-booster titer class, even when adjusted by age and sex:

-- in the group of subjects with pre-booster titer <50 AU/ml (class 1), post-booster titers had a 226-folds increase (CI 95% 112.99-416.17, 4,426-16,093 AU/ml);
-- in the group of subjects with pre-booster titer 50-300 AU/ml (class 2), post-booster titers had a 79-folds increase (CI 95% 73.52-85.39, 13,843-16,023 AU/ml);
-- in the group of subjects with pre-booster titer 301-522 AU/ml (class 3), post-booster titers had a 50-folds increase (CI 95% 47.62-54.28, 19,021-21,634 AU/ml);
-- in the group of subjects with pre-booster titer 523-942 AU/ml (class 4), post-booster titers had a 38-folds increase (CI 95% 35.90-40.80, 25,465-28,925 AU/ml);
-- in the group of subjects with pre-booster titer >942 AU/ml (class 5), post-booster titers had an 18-folds increase (CI 95% 17.14-20.39, 31,968-36,817 AU/ml).

Moreover, the increases in the post-booster titers of classes 1, 3, 4 and 5 significantly differ from the one of class 2. **Table 3S** extensively shows pre- and post-booster GM and CI 95% according to 5 classes of pre-booster titers.

## Discussion

To our knowledge, this is the first study evaluating the antibody response after booster dose in a large population of healthcare workers. In a recent letter, Raz et al. (16) showed a significant increase in anti-S IgG titers after a booster dose administration. Despite a different prevalence of seronegative subjects at the pre-booster evaluation, at least in part explained by the younger age of enrolled subjects, our data are consistent with those reported by Raz et al. regarding the population over 60 years. In our sample, the mean pre- and post-booster titer are slightly lower to that observed in the previous study, but the interval between the second dose and the booster dose was longer, even though in both samples the evaluation was performed 14 days after the booster dose.

A recent claim published by Moderna revealed that at 29 days after the authorized 50 µg booster of mRNA-1273, neutralizing geometric mean titers (GMT) against Omicron were increased of 37 folds with respect to the pre-booster levels (17), similarly to what observed in our study (35 to 53 folds according to classes of age).

We have highlighted a difference in the pre-booster antibody levels and in the mean increase according to a previous exposure to SARS-CoV-2 and to the timing of exposure. To note, since booster dose was recommended by the Italian Ministry of Health only after five months from SARS-CoV-2 infection, in our sample subjects with breakthrough infection after double vaccination were only 12 (0.7%) thus explaining the large 95% confidence interval in the analysis. However, higher pre-booster titers in previously infected compared to SARS-CoV-2-naïve subjects suggest the possibility to defer booster dose according to the infection status. Furthermore, in the very first period of the vaccination campaign, Italian guidelines suggested the vaccination with two doses for non-naïve SARS-CoV-2 subjects as well. However, titers of subjects who were non-naïve for SARS-CoV-2 infection were significantly higher before the booster dose, suggesting the possibility to administer a booster dose later according to the infection status.

Overall, a key result of our study is that the post-booster titer obtained in all subgroups was very similar, independently from pre-exposure history and any other variable.

To overcome the limitation imposed by the presence of an upper superior limit of antibody detection, we have used a Tobit regression model designed to estimate linear relationships between variables when there is censoring in the dependent variable. However, since these limits especially affect the titration results of SARS-CoV-2-naïve subjects, an eventual underestimation would affect mostly this latter category.

The main finding of this work is that the booster dose induced a potent antibody response. This response was independent from sex, while some differences were shown according to age and pre-booster titers. However, the post-booster titer was very high for all the evaluated categories. Other experiences obtained with other vaccines boosters, such as in the case of hepatitis B and tetanus (18, 19), showed a correlation between the pre-booster and the post-booster titer. Relatively to mRNA vaccination, a robust induction of antigen-specific CD4+ and CD8+ T cells operated by COVID-19 vaccination has been demonstrated (20). Vaccine-induced CD4+ T cells are predominantly Th1 and T follicular helper (Tfh) cells, like those generated by SARS-CoV-2 infection. Tfh cells have a key role in plasma cells, antibodies and B memory cells development, while Th1 cells are able to sustain and enhance the quality of memory CD8+ T cell response. This evidence supports the hypothesis that the stimulation of a robust memory response could have elicited a potent antibody production as in the case of our population. Moreover, it has been demonstrated that a second vaccination improved T cell responses in SARS-CoV-2-naive subjects, while it had little effect in SARS-CoV-2-recovered individuals. This could explain a relative lower increase in the antibody titers in the population of previously exposed to SARS-CoV-2 subjects.

The rise in the boosted antibody titer could express the ability of T cells of responding to the entire spike protein by fostering the maturation of broad-spectrum neutralizing antibodies, potentially more effective in neutralizing different viral variants.

These data are important to better understand the COVID-19 vaccination immunization mechanism.

Even though we did not evaluate the incidence subsequent of infection and of disease and despite the lack of a correlate of protection with antibody titer, a higher antibody titer was found to be associated with lower risk of symptomatic disease (21). In agreement with these observations, earlier findings from Israel showed the efficacy of the booster dose in increasing the ability to prevent infection and severe illness: the rate of confirmed infection and of severe illness were lower in the booster group than in the non-booster group by a factor of 11.3 and 19.5, respectively (22, 23). Taken together, we can infer the same effect in our study population.

An important limitation of our study is the lack of neutralizing antibodies measure, however the correlation between the anti-RBD IgG titer and neutralizing antibodies has been described in literature (24).

## Conclusion

Our findings suggest the ability of BNT162b2 to induce a potent booster response after 7-9 months from the first vaccination cycle in a cohort of healthcare workers, potentially reducing the risk of subsequent SARS-CoV-2 infections and disease even considering viral evolution.

## Data Availability Statement

The raw data supporting the conclusions of this article will be made available by the authors, upon request to the corresponding author. The request must be motivated and approved all the authors of this manuscript.

## Author Contributions

AP designed and planned the observational study, conducted and overviewed the study, analyzed data, drafted the initial manuscript, reviewed and edited the final version of the manuscript. AR supported the planning of the study, conducted the study, acquired data, drafted the initial manuscript, reviewed and edited the final version of the manuscript. AS, GG, SA, TC, PS, MMe and SD’O supported the planning of the study, conducted the study and acquired data. MS, OG and SR supported the planning of the study, conducted the study and worked on lab data. VP analyzed data, drafted the initial manuscript, reviewed and edited the final version of the manuscript. SM, CV, DC, MBe and LZ provided support in the planning of the study and provided data. SG, MMo and MBo provided nursing staff and facilities within the hospital during the study and overviewed the final manuscript. MM and MP overviewed and edited the final manuscript. FS designed the study, overviewed and edited the final manuscript.

## Funding

The study was funded by divisional funds from the Infectious Diseases, Clinical Chemistry and Microbiology, and Transfusion Medicine Units of Niguarda Hospital. The Niguarda Hospital administration provided support through the nursing staff and facilities to carry out the procedures of the study. Abbott s.r.l. partially provided SARS-CoV-2 IgG II Quant for anti-RBD IgG evaluation. All authors confirm that they have had full access to all data in the study and accept responsibility to submit for publication.

## Conflict of Interest

Author Valentina Panetta is employed by L’altrastatistica srl – Consultancy & Training, Rome, Italy.

The remaining authors declare that the research was conducted in the absence of any commercial or financial relationships that could be construed as a potential conflict of interest.

## Publisher’s Note

All claims expressed in this article are solely those of the authors and do not necessarily represent those of their affiliated organizations, or those of the publisher, the editors and the reviewers. Any product that may be evaluated in this article, or claim that may be made by its manufacturer, is not guaranteed or endorsed by the publisher.
