# Supplementary material for: Antibody Response to COVID-19 Booster Vaccination in Healthcare Workers
Source: Front Immunol. 2022 May 26;13:872667. doi: 10.3389/fimmu.2022.872667 (PMC9205631; doi:10.3389/fimmu.2022.872667)
Supplement: Supplementary file 1 [file DataSheet_1.docx]

Supplementary Material

# Supplementary Figures and Tables

**1.1 Supplementary Figures**

**Figure 1S.** Post-booster increase according to sex

 
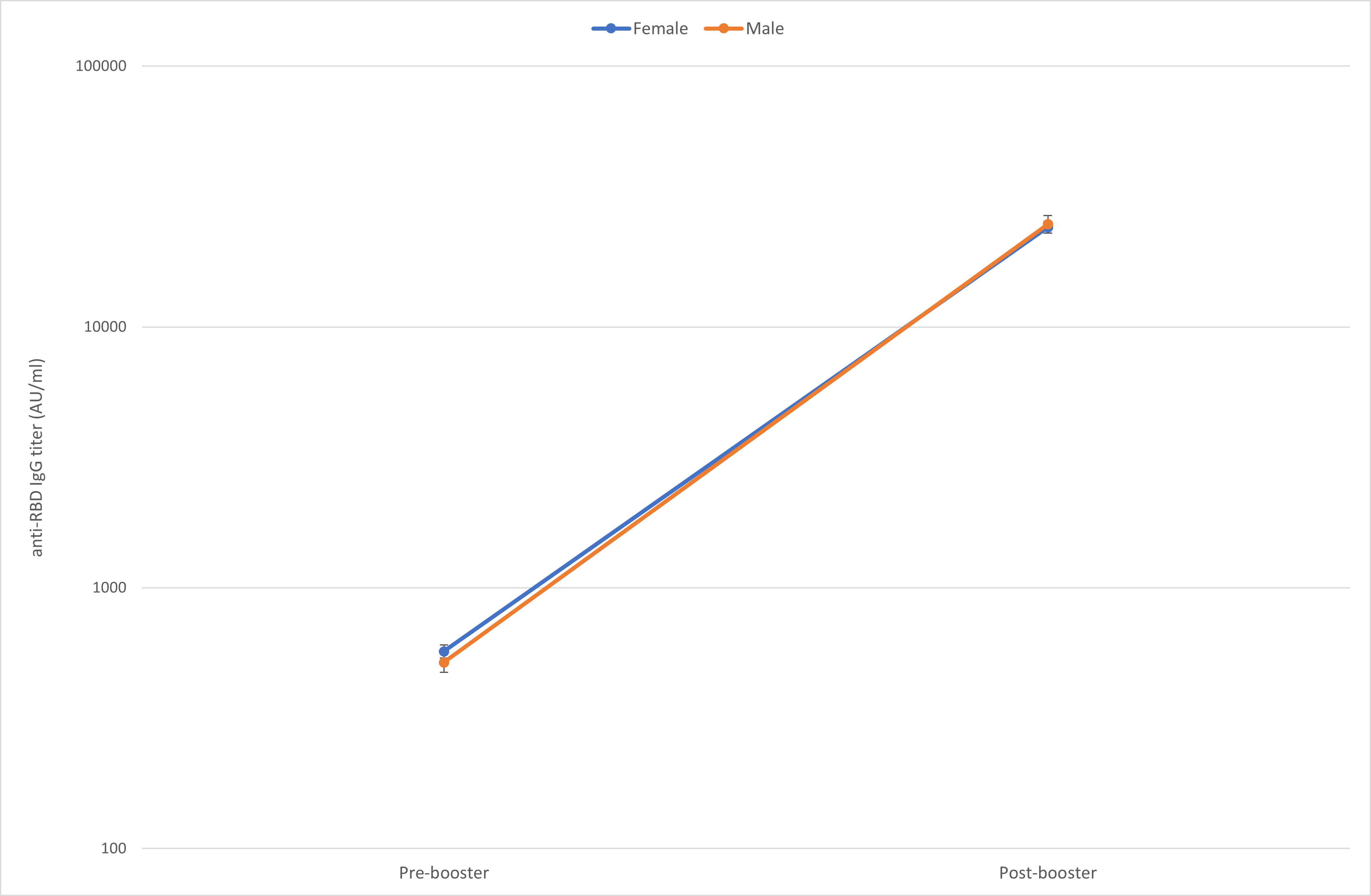


**Figure 2S.** Post-booster increase according to classes of age

 
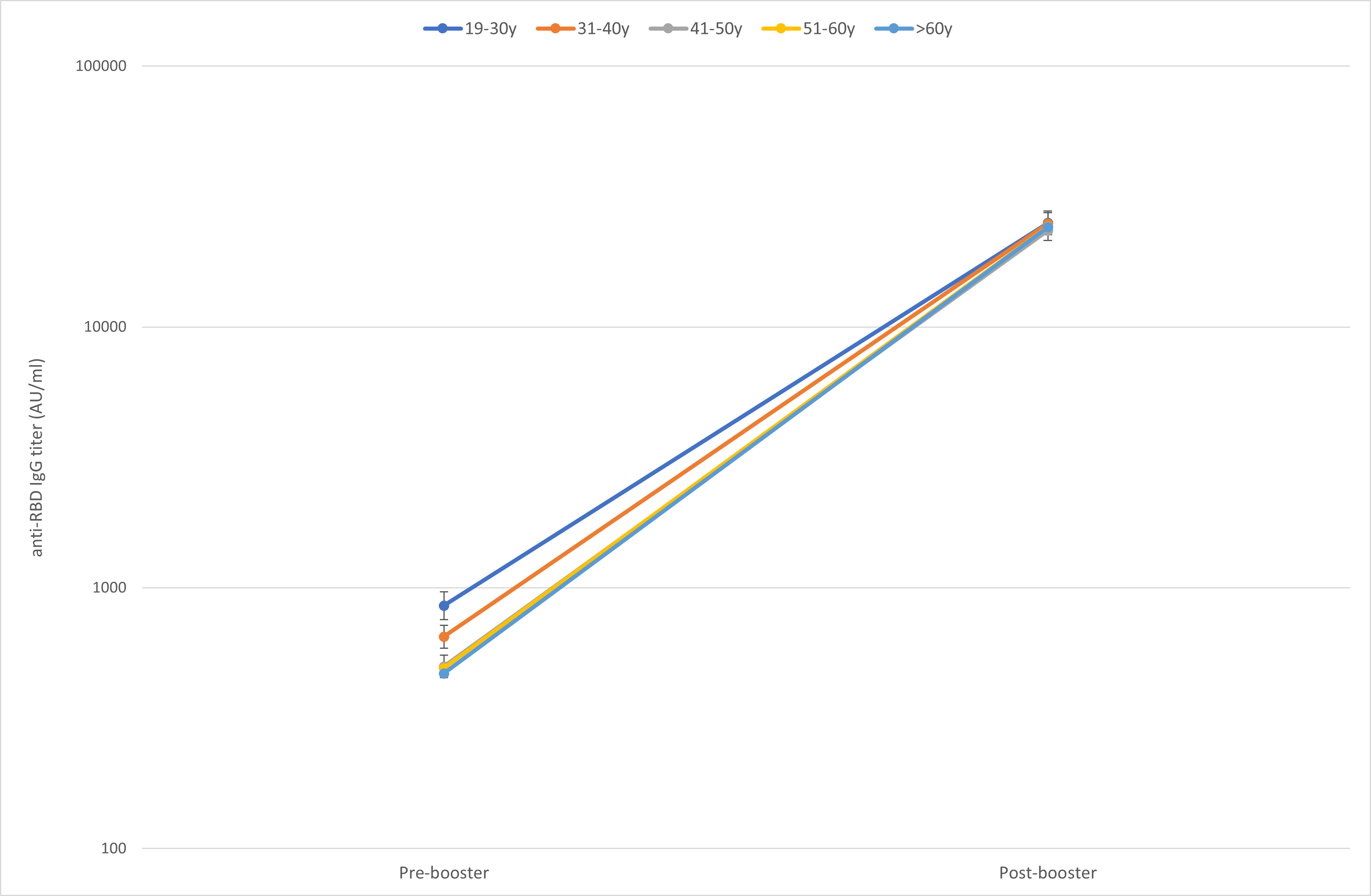


**1.2 Supplementary Tables**

**Table 1S.** Anti-RBD IgG Geometric mean titre before and after booster vaccination according to previous SARS-CoV-2 infection status. GM: geometric mean; CI: confidence interval.

|  | **No SARS-CoV-2 infection** | | | **SARS-CoV-2 infection before vaccine** | | |
| --- | --- | --- | --- | --- | --- | --- |
|  | GM | CI95% | | GM | CI95% | |
| Pre-booster | 555 | 528 | 582 | 1836 | 1603 | 2103 |
| Post-booster | 24447 | 23393 | 25549 | 22236 | 20355 | 24291 |

**Table 2S Anti-RBD Ig Geometric mean titre before and after booster vaccination according to 5 classes of age**

|  | 19-30y | | | 31-40y | | | 41-50y | | | 51-60y | | | >60y | | |  |
| --- | --- | --- | --- | --- | --- | --- | --- | --- | --- | --- | --- | --- | --- | --- | --- | --- |
|  | GM | CI95% | | GM | CI95% | | GM | CI95% | | GM | CI95% | | GM | CI95% | |  |
| Pre-booster | 733 | 640 | 841 | 616 | 559 | 679 | 477 | 433 | 526 | 437 | 437 | 521 | 454 | 393 | 524 | |
| Post-booster | 25675 | 22608 | 29159 | 24952 | 22678 | 27454 | 23563 | 21568 | 25742 | 23870 | 22071 | 25814 | 23891 | 20737 | 27525 | |

**Table 3S** **Anti-RBD IgG Geometric mean titre before and after booster vaccination according to 5 classes of pre-booster titers**

|  | <50 | | | 51-300 | | | 301-522 | | | 523-942 | | | >943 | | |
| --- | --- | --- | --- | --- | --- | --- | --- | --- | --- | --- | --- | --- | --- | --- | --- |
|  | GM | CI95% | | GM | CI95% | | GM | CI95% | | GM | CI95% | | GM | CI95% | |
| Pre-booster | 38 | 32 | 45 | 190 | 183 | 198 | 400 | 393 | 406 | 694 | 694 | 719 | 1831 | 1702 | 1970 |
| Post-booster | 8629 | 4561 | 16325 | 15058 | 13996 | 16200 | 20333 | 19063 | 21689 | 27166 | 25479 | 28964 | 33710 | 31420 | 36167 |
